# Is it possible to rule out level II and level VB dissection in patients with metastatic papillary thyroid cancer?

**DOI:** 10.3389/fendo.2025.1520539

**Published:** 2025-08-11

**Authors:** Tugba Matlim Ozel, Sezer Akbulut, Aykut Celik, Gorkem Yildiz, Hamit Yucel Barut, Fatih Mert Dogukan, Serkan Sari

**Affiliations:** ^1^ Division of Endocrine Surgery, Department of General Surgery, University of Health Sciences Turkey, Basaksehir Cam and Sakura City Hospital, Istanbul, Türkiye; ^2^ Department of Radiology, University of Health Sciences Turkey, Basaksehir Cam and Sakura City Hospital, Istanbul, Türkiye; ^3^ Department of Pathology, University of Health Sciences Turkey, Basaksehir Cam and Sakura City Hospital, Istanbul, Türkiye

**Keywords:** lateral lymph node metastasis, level V dissection, level II dissection, thyroid cancer, lateral lymph node dissection, neck dissection

## Abstract

**Background:**

The completeness of surgical resection is a key factor influencing outcomes in patients with papillary thyroid carcinoma (PTC) and regional lymph node metastases. However, the optimal extent of therapeutic lateral neck dissection remains a matter of debate This study aimed to assess the diagnostic accuracy of preoperative ultrasonography (US) in detecting lateral lymph node metastasis (LLNM) in patients with PTC and to identify clinical and pathological factors predictive of metastases at levels II and V.

**Methods:**

This retrospective study included consecutive patients with PTC who underwent comprehensive lateral neck dissection at a single tertiary center between June 2020 and July 2024.

**Results:**

In 63 patients, a total of 78 comprehensive lateral neck dissections were performed. Of the patients, 41 (65%) were male and 22 (35%) were female, with a median age of 37 years (range, 24–49 years). Lymph node metastases were identified in 46 (58.9%), at level II, 561 (78.2%) at level III, 60 (76.9%) at level IV, and 9 (11.5%) at level Vb. Metastasis to level IIb was detected in 5 dissections. Among the 9 patients with level Vb metastases, 7 (77.8%) had involvement of four different cervical levels. The specificity of US in identifying metastatic disease was notably high at both level II (80%) and level Vb (87%). Independent predictors of metastatic involvement at level II and level Vb lymph nodes was associated with extrathyroidal extension [level II: odds ratio (OR) 7.88, p=0.03; level V: OR 6.91, p=0.043] and a largest metastatic lateral lymph node size above 2 cm [level II: OR 18.58, p=0.03; level V: OR 11.32, p=0.03].

**Conclusion:**

Routine dissection of level IIa is recommended in N1b PTC due to high metastasis rates. However, level IIb dissection may be omitted in selected cases given its low metastasis rate and potential morbidity, with intraoperative frozen section serving as a useful guide. Similarly, level Vb dissection may be avoided when lateral lymph nodes are <2 cm, multilevel involvement is absent, and ultrasonographic findings are negative.

## 1 Introduction

Papillary thyroid carcinoma (PTC) has been increasing in incidence worldwide. Despite this, previous studies have reported 10-year survival rates exceeding 91%, classifying PTC as a low-risk malignancy (1). At diagnosis, regional lymph node metastasis (LNM) is present in approximately 20–90% of patients, most commonly involving the central neck compartment (2, 3). However, LLNM is categorized as N1b disease and is considered more advanced than central LNM (4). Lymph node metastasis in PTC typically begins in the ipsilateral central compartment, followed by involvement of the contralateral central nodes, and then progresses to the ipsilateral lateral neck levels IV, III, IIa, and Vb. Less frequently, metastases may involve levels IIb and Va, and ultimately the contralateral lateral compartment (5). Although rare, skip metastases have also been reported (6). The prevalence of LLNM in PTC ranges from 6.5% to 27.5% in the literature (7). Compared to central compartment metastases, LLNM is associated with a poorer prognosis, including higher recurrence rates, shorter time to recurrence, increased risk of distant metastases, and reduced disease-free survival (8). Among all prognostic factors, the completeness of the initial surgical resection remains the most critical determinant of outcome in patients with PTC. However, there is still controversy regarding the optimal scope of lymph node dissection necessary for effective regional control of LLNM (9).

Historically, surgical management of LLNM has ranged from “berry picking,” which involves selective excision of only grossly involved nodes, to radical neck dissection, which entails en bloc removal of all lymph nodes from levels I to V along with the internal jugular vein, spinal accessory nerve (SAN), and sternocleidomastoid muscle. Due to its high morbidity and disfigurement, radical neck dissection is now rarely performed in thyroid cancer surgery. Currently, to minimize morbidity, particularly because of the low rates of metastasis observed at levels IIb and Va, more limited dissections are recommended for differentiated thyroid cancer metastases. Selective lymph node dissection (SLND), which involves compartment-oriented removal of fewer than five nodal levels, has become the standard surgical technique for therapeutic lymph node (LN) resection in differentiated thyroid cancer (10, 11). In the context of initial SLND for PTC, levels IIa, III, IV, and Vb are commonly included. In this study, the term SLND specifically refers to dissection of these four levels.

However, the extent of this dissection is still controversial. A major question remains as to the therapeutic benefits of a more or less extensive lymphadenectomy and is partly due to the desire to reduce postoperative complications. The more extensive dissection required for LLNM is associated with cosmetic concerns and the potential for nerve injury (SAN, marginal mandibular, phrenic, vagus, hypoglossal, cervical and brachial plexus branches, and ansa cervicalis branches), hemorrhage, and chyle leakage (11). Despite macroscopic preservation of the SAN, patients may experience postoperative shoulder dysfunction and sensory alterations, which can negatively impact quality of life as a result of dissection, traction, or inadvertent transection during level II or Vb lymph node dissection (12). Shoulder dysfunction either temporary or permanent occurs in up to 25–67% of cases and is most commonly attributed to dissection involving level IIb LNs (13). According to previous studies, the metastasis rate in level IIb LNs varies from 2.1% to 22.0% (14, 15). Therefore, routine dissection of level II LNs remains controversial due to the relatively low incidence of metastasis and the high risk of complications, particularly shoulder dysfunction. Similarly, several studies have suggested that routine dissection of level V LNs may not be necessary in patients with LLNM, as the frequency of metastasis and recurrence at this level is relatively low (16, 17), and level V dissection has also been associated with postoperative shoulder dysfunction (12, 18). Conversely, other studies have reported that the incidence of occult LLNM in PTC patients may range from 18.6% to 64% (19, 20), and that over 80% of patients with N1b disease exhibit multilevel LN involvement. These findings highlight the challenge of determining the appropriate extent of dissection (21, 22).

Given the generally favorable prognosis and normal life expectancy of most patients with PTC, minimizing surgical morbidity is a key concern for both patients and clinicians. Preserving level II and V LNs during surgery may help reduce complications associated with LN dissection. Thus, determining the optimal extent of LN dissection is essential to balance the potential surgical risks against the oncological benefits in PTC patients with LLNM. In this context, excluding clinically negative level II or V LNs from routine therapeutic dissection may help prevent unnecessary morbidity. However, more precise criteria are needed to identify patients who may be suitable candidates for limited dissection. Therefore, a thorough understanding of the frequency, distribution patterns, and predictive factors of level II and V metastases is critical in selecting patients who may benefit from a more extensive surgical approach.

The present study aimed to evaluate the diagnostic accuracy of preoperative US in detecting LLNM in patients with PTC. In addition, the histopathological findings from LN dissections and associated clinical variables were analyzed to identify potential predictors of level II and V metastases. Histopathological examination served as the reference standard for comparing the presence of metastasis with findings on preoperative imaging across different neck levels. We hope that our study will contribute to guiding decisions regarding the inclusion of these sites in therapeutic neck dissection for N1b PTC patients, particularly in cases where no clinical evidence of (LLNM) is detected at level II or Vb during preoperative evaluation.

## 2 Materials and methods

### 2.1 Study design

The study was conducted at the Department of General Surgery University of Health Sciences, Basaksehir Cam and Sakura City Hospital. The study was approved by the local ethics committee of the University of Health Sciences, Basaksehir Cam and Sakura City Hospital (KAEK- 08.11.2023.553). Clinical and pathological data from the medical records of consecutive patients who underwent total thyroidectomy with bilateral central node dissection and unilateral/bilateral SLND for N1b PTC between June 2020 and July 2024 were retrospectively reviewed. Of the 431 patients reviewed, 63 patients with confirmed LLNM who underwent a total of 78 lateral neck dissections were included in the final analysis.

All patients included in the study underwent preoperative physical examination, neck ultrasonography (US), and thyroid function tests. In addition, fiberoptic nasopharyngolaryngoscopy was routinely performed to assess vocal cord function. All US evaluations and US-guided fine-needle aspiration (FNA) procedures were conducted by a single experienced radiologist. Preoperative US and FNA cytology were performed for both the primary thyroid nodules and suspicious lateral neck LNs. All patients were diagnosed with PTC in the primary thyroid nodule and one or more LNs in the lateral neck by preoperative FNA. Cytological and histopathological evaluations were carried out by the pathology department Patients with incomplete clinicopathologic data, other types of thyroid carcinoma, those who underwent surgery for recurrent PTC or previous neck surgery because of benign or malignant disease, those with a history of radiation therapy due to other diseases and those with distant metastases at initial presentation were excluded. Clinical, demographic, ultrasonographic, and pathological variables were analyzed to identify potential risk factors associated with LNM at level II or level Vb.

### 2.2 Preoperative imaging and histopathology

Patients with preoperative FNA biopsy results of suspicious for PTC or PTC were evaluated through US for LNM. In patients with suspected LLNM on US, FNA biopsy, including cytological examination and thyroglobulin washout was performed. LNs were characterized according to neck level. Demographic and clinical pathology data, including sex, age, tumor size, tumor type, tumor location, multifocality, bilaterality, capsular invasion, extrathyroidal extension (ETE), and lymphovascular invasion, were analyzed. The tumor location was divided into the superior, middle, inferior portions and the isthmus according to the US results, and the intraoperative findings. Pathology revealed aggressive subtypes, such as tall cell, hobnail, solid, and diffuse sclerosing variants. Extranodal extension (ENE) was defined as tumor spread beyond the lymph node capsule. The total number and size of both harvested and metastatic lymph nodes, presence of ENE, and the neck levels of metastatic LNs were recorded. Metastases at each neck level were compared with corresponding findings on preoperative US.

### 2.3 Surgery

All surgical procedures were performed by a single senior endocrine surgeon in collaboration with one of four other endocrine surgeons at our institution. Intraoperative nerve monitoring was routinely utilized in all cases, not only for the recurrent laryngeal nerve but also for the spinal accessory nerve (SAN) and other relevant nerves. Bilateral central compartment node clearance was performed with particular attention to the identification and preservation of the parathyroid glands. SLND, encompassing levels II-III-IV-Vb, was carried out in a station-by-station manner (16). As a standardized procedure, all patients with LLNM underwent SLND. Dissection of level IIb was carried out selectively in cases where, during intraoperative assessment, the surgeon identified lymph nodes at level IIa that appeared conglomerated or exhibited features suggestive of malignancy. Bilateral SLND was performed in patients with bilateral LLNM. During surgery, neck levels were clearly marked, and lymph node specimens were separated by level before being sent for pathological evaluation. Informed consent was obtained from all patients.

### 2.6 Statistical analyses

SPSS (Statistical Package for the Social Sciences) version 27 software (IBM Corp., Armonk, NY, USA) was used for statistical analysis. Whether the scores obtained from each continuous variable were normally distributed was analyzed via descriptive, graphical and statistical methods. To test the normality of the scores obtained from a continuous variable by statistical methods, the Kolmogorov–Smirnov test was utilized. Categorical variables are presented as frequencies (n, %), and continuous variables are presented as medians and interquartile ranges. Continuous variables were analyzed as categorical variables by considering median and quartile distributions. Chi-square tests (Pearson chi-square test and Fisher’s exact test) were used to investigate variables associated with the presence of level II or V metastasis in univariate analyses. The sensitivity and specificity ratios were calculated for the diagnostic performance of the clinical findings. The agreement between the diagnostic evaluations was measured by kappa (κ) analysis. The level of agreement was considered poor when κ was less than 0, mild-low when 0 ≤ κ ≤ 0.2, moderate when 0.2 < κ ≤ 0.4, acceptable-good when 0.4 < κ ≤ 0.6, significant-high when 0.6 < κ ≤ 0.8 and excellent when κ > 0.8. The independent variables associated with the presence of level II and level V metastases were subjected to multivariate logistic regression model analysis to determine the results. The results were evaluated at the 95% confidence interval, and significance was evaluated at p<0.05 (two-sided).

## 3 Results

### 3.1 Demographic and clinicopathological characteristics

A total of 78 SLND were performed in 63 patients, 48 of which were unilateral and 15 were bilateral. The age of the patients ranged from 24–49 years, with a median age of 37 years and 41 (65%) were male, and 22 (35%) were female.

The median diameter of the primary tumor was 2.1 cm (range 1.3–3.5). Microcarcinoma was found in 5 (8%) patients (**Table 1**). Multifocality, bilaterality, extracapsular invasion, vascular invasion, and ETE were observed in 28 (44.4%), 30 (47.6%), 33 (52.4%), 23 (36.5%), and 15 (23.8%) patients, respectively. The primary tumors were in the superior, middle, inferior, and isthmus regions in 17 (27%), 33 (52.4%), 6 (9.5%), and 7 (11.1%) patients, respectively. Tumor staging based on the T classification was as follows: T1 in 24 patients (38.1%), T2 in 19 patients (30.2%), T3 in 19 patients (30.2%), and T4 in 1 patient (1.6%). Among the 63 patients with N1b PTC, the histological subtypes included classic variant in 37 patients (58.7%), follicular variant in 3 patients (4.8%), and aggressive variants in 23 patients (36.5%). As determined from the dissection samples, the median total numbers of excised lateral LNs and metastatic LNs were 47 (range 22-153) and 5 (range 1-22), respectively. The median size of the largest metastatic LN was 2 cm (range 0.4–5.7 cm). ENE was observed in 27 (42.9%) patients. Skip metastasis was found in 6 patients (9.5%). The detailed demographic and clinicopathologic characteristics of the patients are presented in **Table 1**.

**Table 1:** Demographic and clinicopathological characteristics of N1b PTC patients.

Characteristics (N=63)	Category	n (%)
Age (year)	Median (IQR)	37 (24-49)
Sex	Male	41 (65.1)
	Female	22 (34.9)
Tumor diameter (cm)	Median (IQR)	2.1 (1.3-3.5)
Tumor location	Superior	17 (27)
	Middle	33 (52.4)
	Inferior	6 (9.5)
	Isthmus	7 (11.1)
T staging	pT1	24 (38.1)
	pT2	19 (30.2)
	pT3	19 (30.2)
	pT4	1 (1.6)
Multifocality	Yes	28 (44.4)
	No	35 (55.6)
Bilaterality	Yes	30 (47.6)
	No	33 (52.4)
Extrathyroidal extension	Yes	15 (23.8)
	No	48 (76.2)
Pathologic extranodal extension	Yes	27 (42.9)
	No	36 (57.1)
Macroscopic extranodal extension	Yes	33 (52.4)
	No	30 (47.6)
Capsule invasion	Yes	33 (52.4)
	No	30 (47.6)
Vascular invasion	Yes	23 (36.5)
	No	40 (63.5)
Largest metastatic LN size (lateral region) (cm)	Median (IQR)	2 (0.4-5.7)
Number of metastatic LN (lateral region)	Median (IQR)	5 (1-22)
Number of retrieved LN (lateral region)	Median (IQR)	47 (22-153)
Number of metastatic LN (central region)	Median (IQR)	8 (0-26)
Number of retrieved LN (central region)	Median (IQR)	22 (9-49)

IQR (P25–P75), Interquantile range.

### 3.2 Pattern of lateral lymph node metastasis

Among the initial dissections, 46 (58.9%), 61 (78.2%), 60 (76.9%), and 9 (11.5%) had positive LNs at levels II, III, IV, and Vb, respectively. The rate of multilevel metastasis was 91%. The most common type was two-level metastasis (50%), and the most common metastatic pattern involved three levels: II, III and IV (32%). For 50 of the 78 SLNDs, level IIb dissection was also performed because of the suspicion of LNM at level IIa. However, among the 50 dissections performed due to suspected LNM at level IIa, metastasis at level IIb was detected in 5 (10%) dissections, while metastatic involvement was identified at level IIa in 35 (70.0%) dissections. In 3 dissections, despite the absence of metastasis at level IIa, metastatic lymph nodes were detected at level IIb. Of the nine dissections with level Vb metastases, seven (77.8%) had metastatic involvement at four different cervical levels. (**Table 2**).

**Table 2:** Pattern of lateral lymph node metastasis.

Frequency of metastatic involvement at each level	Number of dissections (n:78)	(%)
Level II	46	58.9
Level IIa	43	53.8
Level IIb	5	7.9
Level III	61	78.2
Level IV	60	76.9
Level Vb	9	11.5
The number of affected levels simultaneously and distribution of metastases		
**1 level only**	**(n=7)**	
Level II/Level III/Level IV	2/2/3	9
**2 levels**	**(n=39)**	
Level II-III	17	21.8
Level III-IV	14	17.9
Level II-IV	6	7.7
Level II-Vb/Level IV-Vb	1/1	1.3/1.3
**3 levels**	**(n=25)**	
Level II-III-IV	25	32
**4 levels**	**(n=7)**	
Level II-III-IV-Vb	7	9

### 3.3 Diagnostic performance of preoperative ultrasonography at individual cervical lymph node levels

A comparison between ultrasonographic and histopathological findings is presented in **Table 3**. The sensitivity of US for detecting metastatic LNs was relatively high at levels III (70%) and IV (75%), but lower at levels II (41.9%) and V (44.4%). Conversely, the specificity of US was higher at levels II (80%) and Vb (87%), and lower at levels III (38.5%) and IV (54.5%). US showed the best pathologic agreement in the detection of level Vb LN involvement (κ=0.288; p=0.021).

**Table 3:** Diagnostic value of preoperative ultrasonography at different cervical levels.

		Ultrasonography			
Neck level	Histopathology (n=63)	Positive	Negative	*κ*	*P* value
II	Positive (43)	18 (41.9%)^a^	25 (58.1%)	0.171	0.090
	Negative (20)	4 (20%)	16 (80%)^b^		
III	Positive (50)	35 (70%)^a^	15 (30%)	0.071	0.559
	Negative (13)	8 (61.5%)	5 (38.5%)^b^		
IV	Positive (52)	39 (75%)^a^	13 (25%)	0.230	0.052
	Negative (11)	5 (45.5%)	6 (54.5%)^b^		
Vb	Positive (9)	4 (44.4%)^a^	5 (55.6%)	**0.288**	0.021*
	Negative (54)	7 (13%)	47 (87%)^b^		

*p<0.05; *κ*, kappa test; a, sensitivity; b, specificity.

### 3.4 Surgical complications

Chyle leakage was observed in 3 (4.7%) patients. All patients who experienced chyle leakage were cured by a fat-restricted diet and local compression. Temporary nerve injury occurred in 6 (7.7%) initial dissections (2 marginal mandibular; 2 branches of the ansa cervicalis and 2 branches of the SAN). There was no permanent nerve injury. Mild shoulder weakness developed in 7 (11%) patients, but full extension was retained, and the weakness improved with physical therapy.

Temporary recurrent nerve injury was observed in 4 (6.3%) patients, and permanent recurrent nerve injury was observed in 1 (1.6%) patient. Temporary hypoparathyroidism was observed in 15 (23%) patients, and permanent hypoparathyroidism was observed in 4 (6.3%) patients. Peroperatively, internal jugular vein injury developed during dissection in 5 patients and was repaired primarily. External jugular vein injury due to invasion developed in 1 patient and was ligated.

### 3.5 Variables predictive of level II or Vb metastases

Univariate analysis revealed that tumors >2 cm (55% vs. 79%; p=0.039), the presence of ETE and pathologic ENE (p=0.024 and p=0.012, respectively), the largest metastatic nodal size (>2 cm metastatic LNs in SLND 54% vs. 89%; p=0.004), macroscopic ENE (assessed by the surgeon) (p<0.001), image-based level II or level V involvement (56% vs. 83%; p=0.022), the number of metastatic LNs above 5 on SLND (42% vs. 87%; p<0.001), and a metastatic LN ratio above 0.1 in the SLND material (48% vs. 85%; p=0.002) were significantly associated with level II metastasis.

Univariate analysis revealed that tumors >2 cm (3.4% vs 23.5%; p=0.031), the presence of ETE and pathologic ENE (p=0.029 and p=0.031, respectively), the largest metastatic nodal size (>2 cm metastatic LNs in the SLND (5.4% vs 27%; p=0.026), and image-based level V involvement (36% vs 9.6%; p=0.042) were significantly associated with level V metastasis (**Table 4**).

**Table 4:** Clinicopathologic variables in relation to level II or Vb metastases.

Variables	Category	All	Patients with level II metastatic (n=43)**		*P* value	Patients with level Vb metastatic (n=9)		*P* value
Age	<40	36	25	(69.4)	0.815^a^	5	(13.9)	>0.999^b^
	≥40	27	18	(66.7)		4	(14.8)	
Sex	Male	41	27	(65.9)	0.576^a^	6	(14.6)	>0.999^b^
	Female	22	16	(72.7)		3	(13.6)	
Tumor size	≤2 cm	29	16	(55.2)	0.039^a^*	1	(3.4)	0.031^b^*
	>2 cm	34	27	(79.4)		8	(23.5)	
Tumor location	Superior	17	12	(70.6)	0.184^b^	4	(23.5)	0.570^b^
	Middle	33	22	(66.7)		4	(12.1)	
	İnferior	6	6	(100)		1	(16.7)	
	Isthmus	7	3	(42.9)		0	(0)	
Multifocality	Yes	28	20	(71.4)	0.628^a^	4	(14.3)	1^b^
	No	35	23	(65.7)		5	(14.3)	
Bilaterality	Yes	30	22	(73.3)	0.409^a^	4	(13.3)	>0.999^b^
	No	33	21	(63.6)		5	(15.2)	
Extrathyroidal extension	Yes	15	14	(93.3)	0.024^b^*	5	(33.3)	0.029^b^*
	No	48	29	(60.4)		4	(8.3)	
Pathologic extranodal extension	Yes	27	23	(85.2)	0.012^a^*	7	(25.9)	0.031*
	No	36	20	(55.6)		2	(5.6)	
Macroscopic extranodal extension	Yes	33	29	(87.9)	<0.001^a^*	7	(21.2)	0.152^b^
	No	30	14	(46.7)		2	(6.7)	
Capsule invasion	Yes	33	22	(66.7)	0.777^a^	4	(12.1)	0.725^b^
	No	30	21	(70)		5	(16.7)	
Vascular invasion	Yes	23	18	(78.3)	0.196^a^	4	(17.4)	0.713^b^
	No	40	25	(62.5)		5	(12.5)	
Largest met node size lateral	≤2 cm	37	20	(54.1)	0.004^a^*	2	(5.4)	0.026^b^*
	>2 cm	26	23	(88.5)		7	(26.9)	
Number of metastatic nodes lateral	1-4	26	11	(42.3)	<0.001^a^*	2	(7.7)	0.286^b^
	≥5	37	32	(86.5)		7	(18.9)	

*p<0.05; ** Metastasis at level II was detected in 43 patients, while a total of 52 dissections were performed at this level. **a**, Pearson chi-square test; **b,** Fisher's exact test.

### 3.6 Multivariate logistic regression for pathological level II or Vb metastasis

To identify risk factors for level II and level Vb LNM in PTC patients, variables with statistically significant differences were included in the multivariate analysis. On multivariate analyses, the independent variables that increased pathologic level II and level Vb LN involvement were ETE [(level II, OR 7.88, 95% CI 1.03–60.59; p=0.03) and level Vb, OR 6.91, 95% CI 1. 06–45.10; p=0.043)] and the largest metastatic nodal size of lateral LNs above 2 cm [level II, OR 18.58, 95% CI 1.33–259.66; p=0.03) and OR, 11.32, 95% CI 1.26–101.59; p=0.03] (p<0.05) (**Table 5**).

**Table 5:** Multivariate logistic regression for pathological level II or Vb metastasis.

	Pathologically level II +			
Variable	β	OR (95% CI)	*P* value	Model
Tumor size (>2 cm)	1.49	4.43 (0.37-52.93)	0.240	χ² =15.684
Largest metastatic node size lateral (>2 cm)	2.92	18.58 (1.33-259.66)	0.030*	p=0.028
Extrathyroidal extension	2.07	7.88 (1.03-60.59)	0.047*	
Pathologic extranodal extension	1.72	5.57 (0.41-76.38)	0.199	
Macroscopic extranodal extension	0.37	1.45 (0.11-19.76)	0.781	

	Pathologically level Vb +			
Variable	β	OR (95% CI)	*P* value	Model
Tumor size (>2 cm)	1.33	3.77 (0.32-43.92)	0.289	χ² =16.03
Largest metastatic node size lateral (>2 cm)	2.43	11.32 (1.26-101.59)	0.030*	p=0.007
Extrathyroidal extension	1.93	6.91 (1.06-45.10)	0.043*	
Pathologic extranodal extension	1.55	4.73 (0.48-46.84)	0.184	

*p<0.05; β, regression estimation value; OR, odds ratio; CI, confidence interval.

## 4 Discussion

The impact of LLNM on survival in patients with PTC remains a matter of debate. Current guidelines (10, 23) and several recent studies (24–26) recommend comprehensive neck dissection, including levels II–V, to achieve optimal disease control in the lateral neck. However, other studies argue against this approach due to the potential for increased morbidity and the lack of demonstrated survival benefit compared to SLND (12, 18, 27). Therefore, the present study was conducted to evaluate the necessity of SLND in patients with N1b PTC and to identify under which conditions more limited dissection may be appropriate.

In this study, we analyzed the pattern of cervical LNM in 63 patients with biopsy-confirmed LLNM. The central compartment was identified as the most frequently involved region among these patients. Skip metastasis was observed in 9.5% of patients, consistent with findings reported in previous studies (2, 3, 21). Therefore, routine clearance of lymph nodes at level VI is justified in patients with N1b PTC, regardless of the clinical suspicion of level VI involvement. Additionally, preoperative evaluation of lateral LNs should be routinely performed, even in the absence of clinically apparent metastasis (11, 19). Levels III and IV were found to be the second most frequently affected regions, with metastasis rates of 79.5% and 82.5%, respectively. This suggests that levels III and IV, in addition to level VI, should be routinely included in neck dissection procedures for N1b PTC patients. According to a study by Javid et al., metastasis was observed in 68.8%, 65.7%, 52.0%, and 16.9% of cases at Levels II, III, IV, and V, respectively. The rate of ipsilateral persistence and recurrence was 10.9%. Additionally, the study reported that among patients who underwent secondary surgery for recurrence, the metastatic lymph node rates at levels II, III, IV, and V were 46.2%, 25.6%, 33.3%, and 12.8%, respectively. Based on these findings, researchers have recommended formal lateral neck dissection, including levels II, III, IV, and V, in patients with PTC, noting that omission of levels II and V may lead to missed metastases in approximately two-thirds of patients at level II and one-fifth at level V (25). They also highlighted that, in high-volume centers, this extended dissection does not increase the rate of permanent nerve injury within the dissected regions. There are different results concerning the extent of LLNM in PTC. Several studies have indicated that levels III and IV are the most frequently involved sites in LLNM, suggesting that targeting these levels is sufficient for the treatment of N1b PTC (17, 26). In a review conducted by Palazzo et al. noted a shift away from extensive neck dissections or berry picking toward SLND (28). On the contrary other studies have found that in patients undergoing lateral neck dissection, the involvement rates for levels II and V LNs are similar to those of levels III and IV. For example, Zhang et al. (29) reported that levels II and V were involved in 49.6% and 20.8% of cases, respectively. Likewise, Liu et al. (21) observed that while levels III and IV were the most commonly metastatic, multilevel metastases were prevalent, with levels II and V showing 43.4% and 27.1% involvement, respectively. In a meta-analysis metastasis rates at levels II, III, and IV reported as 53.4%, 70.5%, and 66.3%, respectively, and a 25.3% rate at level V. Based on these findings, the researchers recommended routine dissection of levels IIa, IIb, III, IV, and Vb in cases of LLNM (30). In addition, a review of 28 studies including patients with differentiated thyroid carcinoma who underwent lateral neck dissection showed that at least 50% of patients had involvement at level II and approximately 20% at level V (31). In this study, the metastatic rates for levels II and Vb (68.3% and 14.3%, respectively) were lower than those observed at levels III, IV, and VI, showing a notably higher rate of level II disease and a lower rate of level Vb disease, as reported in previous studies.

In studies examining the rate of level IIb LNM the frequency of level IIb metastasis has ranged from 2.1% to 22% (14, 15, 22, 32–34). Recent reviews and meta-analyses have reported that 13.7% of level IIb dissections show LNM, which is similar to the 10% incidence found in this study (35, 36). Lee et al. found that 34% of patients with metastatic LNs at level IIa (11 out of 32) also had involvement at level IIb. Additionally, among 12 specimens with metastatic lymph nodes at level IIb, 11 (92%) had metastasis at level IIa. They recommended that level IIb nodes should be included in the dissection whenever metastasis is detected at level IIa, and that dissection of level IIb may not be necessary if level IIa LNs are not involved (37). In a study conducted by Farrag et al. the rates of LNM in N1b PTC patients at levels IIa, IIb, III, IV, Va, and Vb in PTC were 60%, 8.5%, 66%, 50%, 0%, and 40%, respectively. Additionally, the same study reported that all patients with level IIb metastasis also had macroscopic metastasis at level IIa. Based on these findings, the authors have supported routine dissection of levels IIa, III, IV, and Vb in cases of N1b metastatic PTC. Additionally, they recommended to perform level IIb dissection if metastasis at level IIa is confirmed by preoperative FNA or if macroscopic metastatic LNs at level IIA are detected intraoperatively (38). In this study, the rate of level IIa metastasis was found to be 53.8%, consistent with the literature. This relatively high rate underscores the necessity of level IIa dissection. Level IIb dissection was also performed for 50 of the 78 SLND because of suspicion of LNM at level IIa. Metastasis at level IIb was detected in 5 (10%) dissections. In three of the cases with positive level IIb involvement, no metastasis was detected in level IIa. These findings indicated that level IIb dissection should be evaluated independently of level IIa positivity. Therefore, in selected cases without high-risk features such as tumor location, tumor size, extrathyroidal extension, extranodal extension, multilevel metastasis, and large metastatic LNs, level IIb dissection may be omitted or its necessity may be evaluated intraoperatively with the aid of frozen section analysis.

Earlier studies have examined the distribution of LLNM in neck dissections. Liu et al., in their study of 75 consecutive N1b PTC patients, found that 72% exhibited LNM across multiple levels (21). A meta-analysis also indicated that the incidence of multilevel metastasis was 73.9%, and the most frequent pattern is three-level metastasis and levels II, III, and IV pattern affect 27.1% of patients with N1b PTC (6, 22).Similar to the literature, we reported that the rate of multilevel metastasis was 91%, the most common type was two-level metastasis (50%), and the most common metastatic pattern involved three levels: levels II, III and IV (32%).

Some researchers have suggested that dissection of levels II and V may not be necessary if there is no preoperative suspicion of LNM at these levels or if aggressive multilevel LNM is not present (35). However, the sensitivity and negative predictive value of US in assessing the involvement of specific LN levels are relatively low, with sensitivity ranging from 42% to 60% and negative predictive value between 49% and 89% (39).We found that the sensitivity of US alone for detecting LNMs was high for levels III and IV, but comparatively lower for levels II and V and that US showed the best pathologic agreement in the detection of level V LN involvement (κ=0.288; p=0.021). Nevertheless, 60% (25 out of 41) of patients had occult metastasis at level II, while 9.6% (5 out of 52) had occult metastasis at level V. Consistent with our findings, several studies have demonstrated that preoperative imaging has particularly low sensitivity for detecting level V metastases (40). Similarly, a review of 23 studies found that histologically confirmed LLNM was present in 22.5% of level V dissections, but preoperative US was able to detect metastasis in only 2.5% of these cases.

Kim et al. analyzed the outcomes of 646 patients with PTC who underwent lateral neck dissections. They reported a higher rate of shoulder syndrome in patients who underwent routine level V dissection compared with patients who underwent level II-IV dissection (9.1% vs. 2.7%, p=0.002). The researchers recommended routine dissection of levels II–IV in the presence of LLNM. Due to the low risk of metastasis and recurrence to level V, as well as the increased morbidity, they suggested including level V in the dissection only when multilevel metastasis is present at levels II–IV or if metastases are radiologically confirmed at level V (41). In a meta-analysis by Won et al., complication rates based on the extent of lateral neck dissection were examined in 11 studies: they found incidence of shoulder syndrome is significantly less in SLND group compared to extended lateral neck dissections (31).

So far, the appropriate extent of lateral neck dissection for PTC remains uncertain (24, 42). Some studies suggest performing dissection in patients with level IIb LN involvement, despite the relatively low rate of metastasis and the heightened risk of shoulder syndrome (31, 34). Furthermore, the role of level V dissection remains controversial due to low complication rates and lack of significant improvement in survival compared with SLND (31, 41). In this context, alongside US evaluation, the identification of clinical and pathological variables that may assist in predicting level II or V metastasis is crucial. In a study by Merdad et al., younger age and lymphovascular invasion were found as independent predictors of level Vb metastasis, while none of the variables were significantly associated with level II metastasis (43). In another study by Kang et al., the presence of level IV metastasis, macroscopic ENE and primary tumor multifocality were found to be predictive factors for level II and level V metastasis. The authors indicated that dissection of levels III and IV can be carried out in patients with isolated level IV involvement and no macroscopic ENE detected on preoperative US. For patients with level II metastasis, dissection of levels II, III, and IV is recommended when the primary tumor is multifocal or when macroscopic ENE is present. They also suggested that level V dissection might not be necessary in patients who show no level V involvement on preoperative imaging and lack of macroscopic ENE (9).

Several studies have investigated predictive factors associated with level V metastasis. One study identified tumor size ≥2.5 cm, the number of lateral lymph node metastases (LLNMs) ≥3, level III metastases, and the B-type Raf kinase (BRAF) V600E mutation as independent predictors of level V metastasis (44). Zhang et al. identified gross ETE and concurrent multilevel metastasis as independent risk factors for level V LNM. They suggested that including level V dissection is a more rational approach, especially when the primary PTC exhibits these features (24). Koo et al. (32), in their study of 76 patients, found that the simultaneous involvement of levels IIa, III, and IV was an independent predictor of level IIb metastasis. Several studies have shown that level IIb disease occurs more frequently when level IIa is involved (26, 32, 38). In this study, presence of ETE and a largest metastatic lateral LN size above 2 cm were identified as independent predictors of pathological involvement at level II and level Vb. We also observed that although the rate of level IIb metastasis was low, rarely occurred in the absence of level IIa metastasis, and nearly all patients with level Vb metastasis exhibited multilevel LN metastases.

We acknowledge that there were still some limitations in this study. First, our study was nonrandomized and retrospective. Second, our sample size was relatively small, and the disease free and overall survival data were insufficient because of the short-term follow-up period. In the future, a multicenter prospective study should be conducted to evaluate quality of life and survival. Several strengths of this study should be highlighted. First, preoperative LN assessments were performed by the same experienced radiologist, minimizing diagnostic bias related to interobserver variability. Second, all pathological evaluations were conducted by the same group of pathologists, ensuring consistency in the characterization and documentation of PTC features associated LNM. Third, the inclusion of only patients who underwent primary surgery created a homogeneous study population, which enhances the reliability and validity of the study outcomes.

## 5 Conclusion

Compared with other levels in the lateral compartment, levels IIb and Vb exhibit relatively low rates of metastatic lymph node involvement. Given the high metastasis rates reported in several studies, level IIa dissection should be routinely performed in patients with N1b PTC. Given the low incidence of level IIb metastasis and its potential to increase surgical morbidity, routine dissection of this region may be unwarranted. The decision to proceed with level IIb dissection should be individualized, potentially guided by adjunctive diagnostic tools considering patient-specific risk factors. In such cases, intraoperative frozen section evaluation in IIb may be beneficial in guiding the decision to proceed with level IIb dissection. Similarly, level Vb dissection may be omitted when lateral neck lymph nodes are smaller than 2 cm, there is no multilevel involvement, and ultrasonographic findings are negative.

## Data Availability

The datasets presented in this study can be found in online repositories. The names of the repository/repositories and accession number(s) can be found in the article/Supplementary Material.
